# Decreased scene-selective activity within the posterior intraparietal cortex in amblyopic adults

**DOI:** 10.3389/fnins.2025.1527148

**Published:** 2025-02-28

**Authors:** Sarala N. Malladi, Jan Skerswetat, Marianna E. Schmidt, Roger B. H. Tootell, Eric D. Gaier, Peter J. Bex, David G. Hunter, Shahin Nasr

**Affiliations:** ^1^Athinoula A. Martinos Center for Biomedical Imaging, Massachusetts General Hospital, Charlestown, MA, United States; ^2^Department of Psychology, Northeastern University, Boston, MA, United States; ^3^Department of Neurophysics, Max Planck Institute for Human Cognitive and Brain Sciences, Leipzig, Germany; ^4^Max Planck School of Cognition, Leipzig, Germany; ^5^Department of Radiology, Harvard Medical School, Boston, MA, United States; ^6^Department of Ophthalmology, Harvard Medical School, Boston, MA, United States; ^7^Department of Ophthalmology, Boston’s Children Hospital, Boston, MA, United States; ^8^Picower Institute for Learning and Memory, Massachusetts Institute of Technology, Cambridge, MA, United States

**Keywords:** fMRI, amblyopia, scene perception, intraparietal cortex, strabismus, anisometropia, posterior intraparietal gyrus

## Abstract

**Introduction:**

Amblyopia is a developmental disorder associated with reduced performance in visually guided tasks, including binocular navigation within natural environments. To help understand the underlying neurological disorder, we used fMRI to test the impact of amblyopia on the functional organization of scene-selective cortical areas, including the posterior intraparietal gyrus scene-selective (PIGS) area, a recently discovered region that responds selectively to ego-motion within naturalistic environments.

**Methods:**

Nineteen amblyopic adults (10 females) and thirty age-matched controls (15 females) participated in this study. Amblyopic participants spanned a wide range of amblyopia severity, based on their interocular visual acuity difference and stereoacuity. The visual function questionnaire (VFQ-39) was used to assess the participants’ perception of their visual capabilities.

**Results:**

Compared to controls, we found weaker scene-selective activity within the PIGS area in amblyopic individuals. By contrast, the level of scene-selective activity across the occipital place area (OPA), parahippocampal place area (PPA), and retrosplenial cortex (RSC) remained comparable between amblyopic and control participants. The participants’ scores on “general vision” (VFQ-39 subscale) correlated with the level of scene-selective activity in PIGS.

**Discussion:**

These results provide novel and direct evidence for the impact of amblyopia on scene processing within the human brain, thus enabling future studies to potentially link these changes across the spectrum of documented disabilities in amblyopia.

## 1 Introduction

Amblyopia is a developmental disorder caused by disruption of balanced binocular input during early life stages. Amblyopic individuals show reduced visual acuity, typically in one eye, despite normal ocular structure. Amblyopic individuals usually rely on the input from the less affected fellow eye, which enables them to show relatively high binocular visual acuity, comparable to controls, under binocular viewing conditions. However, emerging evidence suggests that amblyopic children and adults show poorer performance during visually guided activities, even when these tasks were conducted binocularly (Birch, 2013; Kelly et al., 2015; Birch et al., 2019; Birch et al., 2022). Among these impairments, limitations in distance vision and peripheral vision especially affect the self-efficacy and quality of life (QoL) for amblyopic individuals.

According to QoL studies, amblyopic individuals have difficulty participating in outdoor physical activities, navigating around objects without collision, and even crossing streets (Kumaran et al., 2019; Randhawa et al., 2023). On one hand, these problems could be due to impairments in depth (McKee et al., 1990; McKee et al., 2003; Levi et al., 2015) and egocentric distance perception either in near personal space (<2 m) (Melmoth and Grant, 2006; Carlton and Kaltenthaler, 2011; Grant and Moseley, 2011) or farther action space (Ooi and He, 2015). According to animal models, these impairments are (at least partly) associated with a decrease in the number of binocularly responsive neurons in V1 (Crawford and Von Noorden, 1979; Horton et al., 1997; Smith et al., 1997a) and decreased sensitivity to binocular disparity within cortical areas V1-V3A (Kumagami et al., 2000; Bi et al., 2011).

On the other hand, difficulties in navigation and visually guided activities could be also due to impaired scene perception in amblyopic individuals. Mirabella et al., have shown that amblyopic individuals show poorer scene discrimination performance and this impairment is detectable even when 2D scene images are perceived with the fellow eye, ruling out the possibility that the poorer scene discrimination performance is due to the monocular impacts of amblyopia (Mirabella et al., 2011). Although the impact of amblyopia on higher order visual areas is expected (Lerner et al., 2003; Muckli et al., 2006), no previous studies have tested the impact of amblyopia on those regions that are selectively involved in scene perception.

In humans, there is a network of visual areas that shows a selectively higher response to scenes, when compared to other visual object categories (Nasr et al., 2011; Kennedy et al., 2024). In this study, we test the hypothesis that amblyopia preferentially impacts function in one or more of these scene-selective area(s). These areas include (but are not limited to): (*i*) the temporal place area known as the parahippocampal place area (PPA) (Epstein and Kanwisher, 1998), (*ii*) the occipital place area (OPA) (Grill-Spector, 2003; Dilks et al., 2013), (*iii*) the medial place area located near the retrosplenial cortex (RSC) (Maguire, 2001; Park and Chun, 2009), and (*iv*) the posterior intraparietal gyrus scene-selective area (PIGS). Compared to other category-selective visual areas, activity within the scene-selective areas relies heavily on the distance between the visual objects and the observer (Kravitz et al., 2011; Persichetti and Dilks, 2016; Park and Park, 2020). Among these areas, PIGS (Kennedy et al., 2024) and OPA (Kamps et al., 2016; Jones et al., 2023) also respond selectively to ego-motion within naturalistic scenes. Thus, considering impairments in distance and ego-motion perception among amblyopic individuals, we expected the amblyopia impact to be stronger on the scene-selective areas, especially in PIGS and OPA.

To test the hypothesis that amblyopia influences the function of scene-selective areas, we first tested (and confirmed) previous reports that amblyopic individuals self-report lower scores for general vision, distance activities, and peripheral vision, compared to age-matched controls (Kumaran et al., 2019; Randhawa et al., 2023). To achieve this goal, we administered the visual function questionnaire (NEI-VFQ 39), a test designed to evaluate the functional and psychological effects of visual conditions such as amblyopia (Mangione et al., 2001). We then tested the hypothesis that amblyopia influences the function of scene-selective areas. Specifically, we used fMRI to compare the evoked scene-selective activity between amblyopic individuals and controls. The fMRI results were compared relative to the individual’s self-reported visual functions to clarify whether or not the activity with scene-selective areas is correlated with the participant’s self-reported performance in daily activities. As a control, to test whether the impact of amblyopia is limited to scenes or if it also affects the response to other stimulus categories, we also measured the level of object-activity in our participants. To reduce the impact of decreased stereoacuity (a common impairment among amblyopic individuals) on the evoked brain response, participants were presented with binocular, 2D images during the fMRI scans. Results of these scans showed a decreased scene-selective (but not object-selective) activity in area PIGS (but not the other scene-selective areas) in amblyopic individuals compared to controls.

## 2 Methods

### 2.1 Participants

Forty-nine adult humans, aged 18–56 years, participated in this study. Among them, nineteen individuals (10 females) were diagnosed with amblyopia. They were either identified through their medical records and invited to participate in the study by their ophthalmologists or learned about the study through word of mouth from friends and colleagues. The remaining thirty participants (15 females) had normal corrected visual acuity in both eyes. They were recruited in response to the study flyer. For both groups, the fMRI study marked their first interaction with the research team.

All participants had radiologically intact brains, without any history of neuropsychological disorder. All experimental procedures conformed to NIH guidelines and were approved by Massachusetts General Hospital protocols. Written informed consent was obtained from all participants before the experiments.

### 2.2 General procedure

The study consisted of a behavioral experiment and two neuroimaging tests. The behavioral tests were performed outside of the scanner, including (1) answering a questionnaire, and (2) conducting ophthalmological assessments. The neuroimaging experiments were conducted on a different day relative to the behavioral tests, inside of a 3 T scanner. The amblyopic participants were scanned while wearing either corrective lenses or MR compatible goggles (i.e., with their best corrected visual acuity). As demonstrated in Table 1, a subset of volunteers (2 controls and 14 amblyopic individuals) participated in all experiments. The others participated in 1 or 2 experiments, depending on their availability at the time.

**Table 1:** Participants demography and contributions.

ID^a^	Age^b^	Gender^c^	Exp 1	Exp 2	Exp 3	ID	Age	Sex	Exp 1	Exp 2	Exp 3
Controls						Controls					
C1	24	M	1	1	1	C16	31	F	0	1	1
C2	30	M	1	1	1	C17	28	M	0	1	1
C3	26	F	1	1	0	C18	30	M	0	1	1
C4	32	M	1	1	0	C19	26	F	0	1	1
C5	22	F	1	1	0	C20	22	M	0	1	1
C6	43	M	1	0	0	C21	25	F	0	1	1
C7	38	F	1	0	0	C22	28	F	0	1	1
C8	38	F	1	0	0	C23	30	M	0	1	1
C9	35	M	1	0	0	C24	32	F	0	1	1
C10	26	M	1	0	0	C25	30	F	0	1	1
C11	23	F	1	0	0	C26	25	F	0	1	1
C12	34	F	0	1	1	C27	40	F	0	1	0
C13	32	F	0	1	1	C28	26	M	0	1	0
C14	38	M	0	1	1	C29	29	M	0	1	0
C15	37	M	0	1	1	C30	27	M	0	1	0
Strabismic						Anisometropic / Deprivational					
S1	40	F	1	1	1	A1	31	F	1	1	1
S2	20	M	1	1	1	A2	23	M	1	1	1
S3	28	M	1	1	1	A3	35	M	1	1	1
S4	26	F	1	1	1	A4	20	F	1	1	1
S5	31	F	1	1	1	A5	26	M	1	1	1
S6	26	F	1	1	1	A6	19	F	1	1	1
S7	21	F	1	1	1	A7	24	F	1	1	1
S8	56	M	1	0	0	A8	26	M	0	1	1
S9	28	M	0	1	1	A9	18	F	0	1	1
						D1	30	M	1	1	1

a C = control, S = strabismic, A = anisometropic, D = deprivation.

b Age is measured in year.

c F=Female, M = Male.

#### 2.2.1 Experiment 1 – behavioral tests

Among the participants, sixteen amblyopic individuals (9 females) and eleven controls (5 females) participated in the behavioral experiment (Table 1). This experiment consisted of two parts: The first part was based on the National Eye Institute (NEI) visual function questionnaire (NEI-VFQ 39), which is a well-validated questionnaire on visual function and disabilities, including subscales on general vision, ocular pain, near vision, distance vision, vision-specific social function, vision-specific mental health, vision-specific role function, dependency, driving, peripheral vision, and color vision. This questionnaire evaluates the subjective experiences of individuals with visual problems, including their ability to perform daily tasks, their emotional well-being, and their overall quality of life.

The second set of behavioral measurements included ophthalmological tests that were conducted outside of the scanner by an optometrist (JS) with extensive experience evaluating amblyopic individuals. Those measurements assessed each participant’s best-corrected monocular and binocular distance visual acuities [ETDRS retro luminant chart (Precision Vision)], the presence of peripheral monocular suppression (Worth 4-dot at near), and stereoacuity [Randot stereo test (Stereo Optical)].

#### 2.2.2 Experiment 2 – scene-selective activity measurement

**Figure 1:**
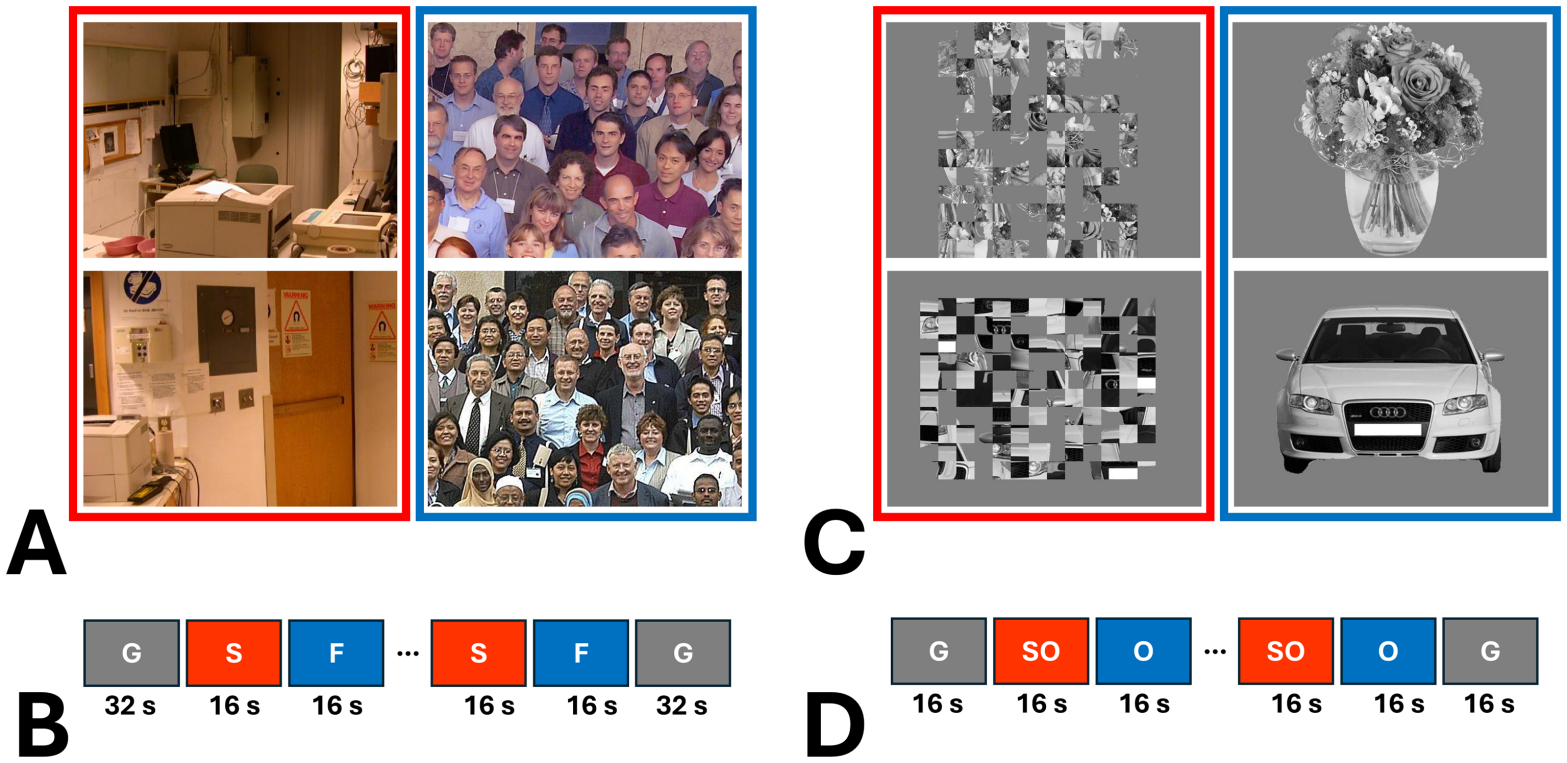
Examples of the stimuli used in this study and a schematic representation of stimulus presentation. **(A)** shows two examples of scenes (S) and group faces (F) used in Experiment 2 (Nasr et al., 2011; Kennedy et al., 2024). As shown in **(B)**, each experiment run consisted of 10 blocks, with an additional 32 s of blank gray (G) presentation at the beginning and end of each run. **(C)** provides examples of objects (O) and scrambled objects (SO) used in Experiment 2. **(D)** illustrates that, in Experiment 3, each run also consisted of 10 blocks, but with 16 s of blank gray presentation at the beginning and end.

Eighteen amblyopic individuals, plus twenty-four controls, participated in this fMRI experiment. During the MRI scans, participants were presented binocularly with 8 naturally colored images of real-world scenes vs. group faces (Figure 1A) (Nasr et al., 2011; Kennedy et al., 2024). Scene and face stimuli were retinotopically centered and subtended 20° × 26° of visual field, without any significant differences between their root mean square (RMS) contrast (t(14) =1.10, *p* = 0.29). Notably, the group face stimuli contained significantly stronger high spatial frequency components (>5 cycles/deg) compared to scenes (*p* = 0.02), ruling out the possibility that scene-selective activity is primarily evoked by this feature (Rajimehr et al., 2011). The levels of low spatial frequency components (<5 cycles/deg) were statistically equivalent between scenes and group faces (*p* = 0.66).

Scene and face stimuli were presented in different blocks (16 s per block and 1 s per image). Each individual participated in 6 runs. Each run consisted of 10 blocks, plus 32 s of a blank gray presentation (used as the baseline) at the beginning and at the end of each run (Figure 1B). Within each run, the sequence of blocks and images within each block were randomized. Data from one amblyopic participant was excluded due to a technical problem in stimulus presentation.

During the scan, stimuli were presented via a projector (1,024 × 768 pixel resolution, 60 Hz refresh rate) onto a rear-projection screen. Participants viewed the stimuli through a mirror mounted on the receive coil array. To ensure that participants were attending the screen, each participant was instructed to report color changes (red to blue and vice versa) of a centrally presented fixation point (0.1° × 0.1°) by pressing a key on the keypad. Participant’s detection accuracy remained above 75% and showed no significant difference in color change detection performance across experimental conditions (*p* > 0.10). MATLAB (MathWorks 2023a; Natick, MA, United States) and the Psychophysics Toolbox (Brainard, 1997; Pelli, 1997) were used to control stimulus presentation.

#### 2.2.3 Experiment 3 – object-selective activity measurement

Among those who participated in Experiment 2, eighteen amblyopic individuals and seventeen controls (9 females) agreed to be scanned further to measure their response to intact and scrambled objects. Stimuli consisted of 38 gray-scale images of intact everyday objects (e.g., tools, furniture and fruits) and their scrambled versions (i.e., no RMS contrast difference) (Figure 1C) (Nasr et al., 2013; Yue et al., 2013). Stimuli were retinotopically centered on a fixation spot and subtended 20° × 20° of visual field. Intact and scrambled images were presented binocularly in different blocks (16 s per block and 1 s per image). Each individual participated in 6 runs, and each run consisted of 8 blocks plus 16 s of blank gray presentation at the beginning and at the end of each block (Figure 1D). Within each run, the sequences of blocks (and images within blocks) were randomized. Other details of the stimulus presentation and the participant’s task during the experiments were identical to Experiment 2.

### 2.3 Imaging

Participants were scanned in a horizontal 3 T scanner (Tim Trio, Siemens Healthcare, Erlangen, Germany). Gradient echo EPI sequences were used for functional imaging. Functional data were acquired using single-shot gradient echo EPI, using isotropic voxels, nominally 3.0 mm on each side (TR = 2000 ms; TE = 30 ms; flip angle = 90°; band width (BW) = 2,298 Hz/pix; echo-spacing = 0.5 ms; no partial Fourier; 33 axial slices covering the entire brain; and no acceleration). During the scan session, structural (anatomical) data were also acquired for each participant using a 3D T1-weighted MPRAGE sequence (TR = 2,530 ms; TE = 3.39 ms; TI = 1,100 ms; flip angle = 7°; BW = 200 Hz/pix; echo-spacing = 8.2 ms; voxel size = 1.0 × 1.0 × 1.33 mm).

### 2.4 Data analysis

Structural and functional data analysis were conducted based on using FreeSurfer (Fischl, 2012).

#### 2.4.1 Structural data analysis

For each participant, inflated and flattened cortical surfaces were reconstructed based on the high-resolution anatomical data (Dale et al., 1999; Fischl et al., 1999; Fischl et al., 2002).

#### 2.4.2 Individual-level functional data analysis

All functional data were rigidly aligned (6 df) relative to participant’s own structural scan, using rigid Boundary-Based Registration (Greve and Fischl, 2009), followed by motion correction. The resulting data was spatially smoothed using a 3D Gaussian kernel (2 mm FWHM). Subsequently, a standard hemodynamic model based on a gamma function was fitted to the fMRI signal, sampled from the middle of cortical gray matter (defined for each participant based on their structural scan), to estimate the amplitude of the BOLD response. Finally, vertex-wise statistical tests were conducted by computing contrasts based on a univariate general linear model (Friston et al., 1999). For presentation of activity maps based on individual participants, the resultant significance maps were projected onto a common human brain template [fsaverage; (Fischl, 2012)].

#### 2.4.3 Group-level functional data analysis

To generate group-averaged maps, functional maps were spatially normalized across participants, then averaged using random-effects models and corrected for multiple comparisons (Friston et al., 1999). The resultant significance maps were projected onto the fsaverage template.

#### 2.4.4 Vertex-wise between-groups comparison

Unless otherwise indicated, between-groups (amblyopic vs. control participants) activity difference maps were also generated based on a random-effects model, after correcting for multiple comparisons.

#### 2.4.5 Region of interest analysis

The main ROIs included area PIGS, plus each of the three previously known scene-selective areas (PPA, RSC, and OPA). We used two separate methods to define the ROIs. The results of using these ROIs were analyzed and reported separately.

##### 2.4.5.1 Labels generated from other datasets

We defined the scene-selective areas based on an independent set of stimuli, other than those used to activate these areas. For this purpose, PIGS was defined based on the group-averaged response evoked by scene>face in a separate group of subjects (Steel et al., 2023). Areas PPA, RSC and OPA were defined based on the dataset of different group of participants with intact vision who were presented with scenes and non-scene objects (including faces, tools, furniture and other daily base objects) (Nasr and Rosas, 2016).

In addition to the scene-selective areas, area V6 (Pitzalis et al., 2010) was also localized using a probabilistic label generated previously based on an independent group of individuals (Kennedy et al., 2024). Notably, this area also shows a significant level of scene-selective response (Sulpizio et al., 2020; Kennedy et al., 2024).

##### 2.4.5.2 Labels generated from odd vs. even runs

Since the independently generated ROIs (see above) were based on the data from individuals with normal vision, it could be argued that our method may be more suitable for measuring the activity in controls rather than amblyopic participants. To answer this concern, a separate set of ROIs were defined independently for amblyopic and control individuals, based on the corresponding group-averaged “scene > face” activity map (based on random-effects and after correction for multiple comparisons). This process was done independently for the odd and even runs for controls (at *p <* 10^−2^ threshold) and amblyopic participants (at *p <* 0.05 threshold). The ROIs that were defined based on the odd runs were used to measure the level of activity evoked during the even runs, and vice versa. This procedure assured us that the ROIs were defined based on a different dataset compared to the test data.

In Experiment 3, we also used the lateral object-selective complex [LOC; (Grill-Spector et al., 2001)] as a control ROI. Area LOC was localized functionally, based on the group-averaged activity map evoked in response to the “intact>scrambled objects” functional contrast. Here again, ROIs were defined for each group, based on their own group-averaged activity maps.

#### 2.4.6 Comparing the size of scene-selective areas

To compare the size of scene-selective areas between amblyopic individuals and controls, these areas were localized for each participant based on their own scene-selective activity map at a threshold of *p* < 10^−2^. These measurements were then normalized relative to the size of the entire cerebral cortex. This procedure assured us that our tests were not confounded by differences in overall brain size.

### 2.5 Statistical tests

To test the effect of independent parameters, we applied paired t-tests and/or a repeated-measures ANOVA, with Greenhouse–Geisser correction whenever the sphericity assumption was violated. The effect of group was tested by comparing the response from controls vs. amblyopic individuals, irrespective of the amblyopia sub-type, unless otherwise is noted. All results were corrected for multiple comparisons.

### 2.6 Data sharing statement

All data, codes and stimuli are ready to be shared upon request.

MATLAB (RRID: SCR_001622; https://www.mathworks.com).

FreeSurfer (RRID: SCR_001847; https://surfer.nmr.mgh.harvard.edu/fswiki/FsFast).

Psychophysics Toolbox (RRID:SCR_002881; http://psychtoolbox.org/docs/Psychtoolbox).

## 3 Results

### 3.1 Participants age and gender distribution

Nineteen amblyopic individuals (10 females), aged 18–56 years, and thirty controls (15 females) with normal or corrected-to-normal visual acuity, aged 22–43 years, participated in this study (Table 1). Amblyopic participants consisted of 9 individuals with strabismus, 9 with anisometropia, and 1 with deprivational amblyopia. None of the participants had combined strabismic and anisometropic amblyopia. Independent applications of t-tests did not yield any significant age differences between amblyopic vs. control participants (t(48) = 1.18, *p* = 0.25) or between anisometropic vs. strabismic participants (t(17) = 1.38, *p* = 0.19). Application of this analysis to the subset of participants who participated in each experiment yielded the same result. Thus, potential differences between groups could not be attributed solely to age differences.

### 3.2 Experiment 1 – ophthalmologic and VFQ-39 tests

Sixteen amblyopic individuals and 11 controls were examined by an optometrist with extensive experience with amblyopia to measure their monocular and binocular visual acuity, monocular suppression, and stereoacuity. They also answered the VFQ-39 questionnaire (see Methods and Table 1).

#### 3.2.1 Ophthalmological assessment

All amblyopic individuals (except for one) showed evidence for either monocular suppression or diplopia (Worth 4-Dot; Table 2). In contrast, all tested controls showed binocular fusion. Moreover, compared to controls, amblyopic individuals showed a significantly higher interocular visual acuity difference (t(25) = 3.15; *p* < 0.01) and poorer stereoacuity (t(25) = 2.52; *p* < 0.01), as expected. However, binocular visual acuity did not differ significantly between the two groups (t(25) = 1.27; *p* = 0.22). Amblyopia severity as determined by the interocular visual acuity difference was comparable between the anisometropic (*n* = 7) and strabismic (*n* = 8) individuals (t(13) = 1.61; *p* = 0.13). Consistent with previous reports (McKee et al., 2003; Levi et al., 2015), strabismic individuals demonstrated more severely impaired stereoacuity (>500 arc seconds) than anisometropic individuals. However, stereoacuity was statistically comparable between anisometropic and strabismic participants (t(13) = 1.57; *p* = 0.14).

**Table 2:** Ophthalmologic assessment of all participants.

ID^a^	Age of diagnosis	Right eye visual acuity^b^	Left eye visual acuity	Binocular visual acuity	Fellow/dominant eye	Suppression worth 4dots	Randot Stereoacuity^c^	Angle of strabismus (at 4 m in PD)
S1	<1	−0.06	+0.06	−0.06	RE	Diplopia	>500	16/25
S2	3	+0.09	+0.30	+0.09	LE	RE	>500	12/10
S3	6	+0.48	−0.02	+0.02	LE	RE	>500	25/18
S4	6	+0.00	−0.06	+0.00	LE	Diplopia	50	4/4
S5	3	+0.26	+0.04	+0.04	LE	RE	>500	10/8
S6	2	+0.46	−0.06	−0.14	LE	RE	>500	10/8
S7	4	−0.08	−0.10	−0.08	LE	Diplopia	70	20/20
S8	5	−0.20	+0.06	+0.06	RE	LE	>500	16/16
S9	2	+0.00	+0.60	+0.00	RE	LE	>500	14/10
A1	5	−0.22	+0.20	−0.16	RE	None	>500	None
A2	5	−0.08	+0.30	−0.04	RE	None	400	None
A3	11	−0.04	+0.26	−0.04	RE	LE	200	None
A4	6	+0.06	+0.32	+0.00	RE	None	40	None
A5	8	+0.00	+0.17	+0.00	RE	Diplopia	200	None
A6	5	+1.00	−0.08	−0.08	LE	RE	>500	None
A7	8	+0.64	−0.10	−0.10	LE	RE	>500	None
A8	6	−1.25	−0.25	−0.25	RE	LE	>500	None
A9	7	+0.40	+0.00	+0.00	RE	None	>500	None
D1	4	−0.26	+0.10	−0.26	RE	LE	100	None

a C = control, S = strabismic, A = anisometropic, D = deprivation.

b Visual acuity is measured in logmar.

c Randot stereoacuity is measured in seconds of arc.

#### 3.2.2 Qualitative assessment of amblyopia impacts on visual capabilities

Previous studies reported that amblyopic individuals struggle with distance activities and peripheral vision (Kumaran et al., 2019; Randhawa et al., 2023). To directly test for analogous results in our cohort, participants in Experiment 1 received the VFQ-39 questionnaire. Consistent with previous studies, we found significantly lower (i.e., worse) scores for amblyopic individuals compared to controls in the general vision, distance activities, and peripheral vision categories (*p* < 0.01; Table 3). In contrast, we did not find any significant difference between the two groups in near activities, color vision, or driving capabilities (*p ≥* 0.08). No significant differences were found between the strabismic and anisometropic individuals across the VFQ-39 subscales (*p ≥* 0.14).

**Table 3:** VFQ-39 subscales.

Subscale		Controls (*n* = 11) (mean ± S.D.)	Strabismic (*n* = 8) (mean ± S.D.)	Anisometric (*n* = 8) (mean ± S.D.)	Control vs. amblyopic participants^a^ (*p*-value)
General health		**88.18 ± 12.50**	**75.31 ± 9.20**	**77.81 ± 15.09**	0.02 *
General vision		**85.45 ± 10.83**	**69.38 ± 16.57**	**66.25 ± 9.16**	**<10**^**−3**^ **
Ocular pain		**94.32 ± 10.25**	**90.62 ± 18.60**	**87.50 ± 11.57**	**0.32**
Near vision		**98.86 ± 3.77**	**97.40 ± 4.42**	**92.71 ± 7.30**	**0.09**
Distance vision		**97.35 ± 5.36**	**86.98 ± 13.63**	**86.98 ± 8.16**	<0.01 **
Vision Specific	Social functioning	**100.00 ± 0.00**	**95.83 ± 8.91**	**98.96 ± 2.95**	**0.21**
	Mental health	**97.27 ± 3.44**	**86.25 ± 19.41**	**83.75 ± 12.17**	0.02 *
	Role difficulties	**100.00 ± 0.00**	**92.97 ± 9.70**	**93.75 ± 4.72**	<0.01 **
	Dependency	**98.86 ± 3.77**	**97.66 ± 4.65**	**100.00 ± 0.00**	**0.98**
Driving		**84.72 ± 13.66**	**77.08 ± 21.53**	**73.44 ± 14.07**	**0.16**
Color vision		**100.00 ± 0.00**	**100.00 ± 0.00**	**100.00 ± 0.00**	**1.00**
Peripheral vision		**97.73 ± 7.54**	**81.25 ± 22.16**	**84.38 ± 12.94**	0.01 **

a The effect of group was tested between controls vs. amblyopic individuals, irrespective of their amblyopia sub-type. A separate test did not show any significant differences between anisometropic and strabismic individuals (not shown here).

*: *p* < 0.05; **: *p* ≤ 0.01.

Beyond the scope of this study, we also found that amblyopic participants reported lower “General Health” (*p* = 0.02), “Mental Health” (*p* = 0.02), plus “Role Difficulties” (*p* < 0.01). These results appear to be consistent with previous findings that amblyopic individuals may show other psychosocial (Satterfield et al., 1993; Packwood et al., 1999; Haine et al., 2025) and health-related (Wagner et al., 2024) problems.

#### 3.2.3 Predictability of VFQ-39 scores based on the ophthalmological measurements

We tested whether the VFQ-39 scores for general vision, distance activities and peripheral vision were predictable based on the level of interocular visual acuity difference, binocular visual acuity and stereoacuity. Among these VFQ-39 subscales, separate Pearson correlation tests showed a significant linear relationship between the interocular visual acuity difference and general vision (df = 25, R^2^ = 0.28; *p* < 0.01), and peripheral vision (df = 25, R^2^ = 0.17; *p* = 0.03). We also found a marginal (statistically non-significant) correlation between the binocular visual acuity and general vision (df = 25, R^2^ = 0.13; *p* = 0.06). The correlations between the other factors were non-significant (df = 25, R^2^ < 0.10; *p* > 0.11). Notably, the correlation between general vision and interocular visual acuity is at least partly driven by the between groups (amblyopic vs. control participants) differences in these two measures and application of the same test just to the results from the amblyopic individuals did not yield any significant correlation between the parameters (*p* > 0.10). More amblyopic individuals with a wide range of visual acuities are required to test this hypothesis more thoroughly in the future.

### 3.3 Experiment 2 – scene-selective cortical response

Experiment 2 was designed to test whether amblyopia is associated with a decrease in the amplitude of scene-selective responses. Eighteen amblyopic individuals, plus twenty-four controls, participated in this experiment (Table 1) and were presented binocularly with scene and face stimuli in different blocks (see Methods).

#### 3.3.1 Head position stability

Head motion has a strong impact on the fMRI signal, and it may influence the level and pattern of evoked fMRI responses, which might thus confound between-groups comparisons. However, a t-test applied to the measured level of head motion did not yield a significant difference between the two groups (t(40) = 1.58, *p* = 0.12). Thus, head motion was statistically comparable across the two groups. Nevertheless, head motion was included as a nuisance covariate in all analyses, to reduce any residual impact of head motion on our findings.

#### 3.3.2 Group-level localization of scene-selective areas

**Figure 2:**
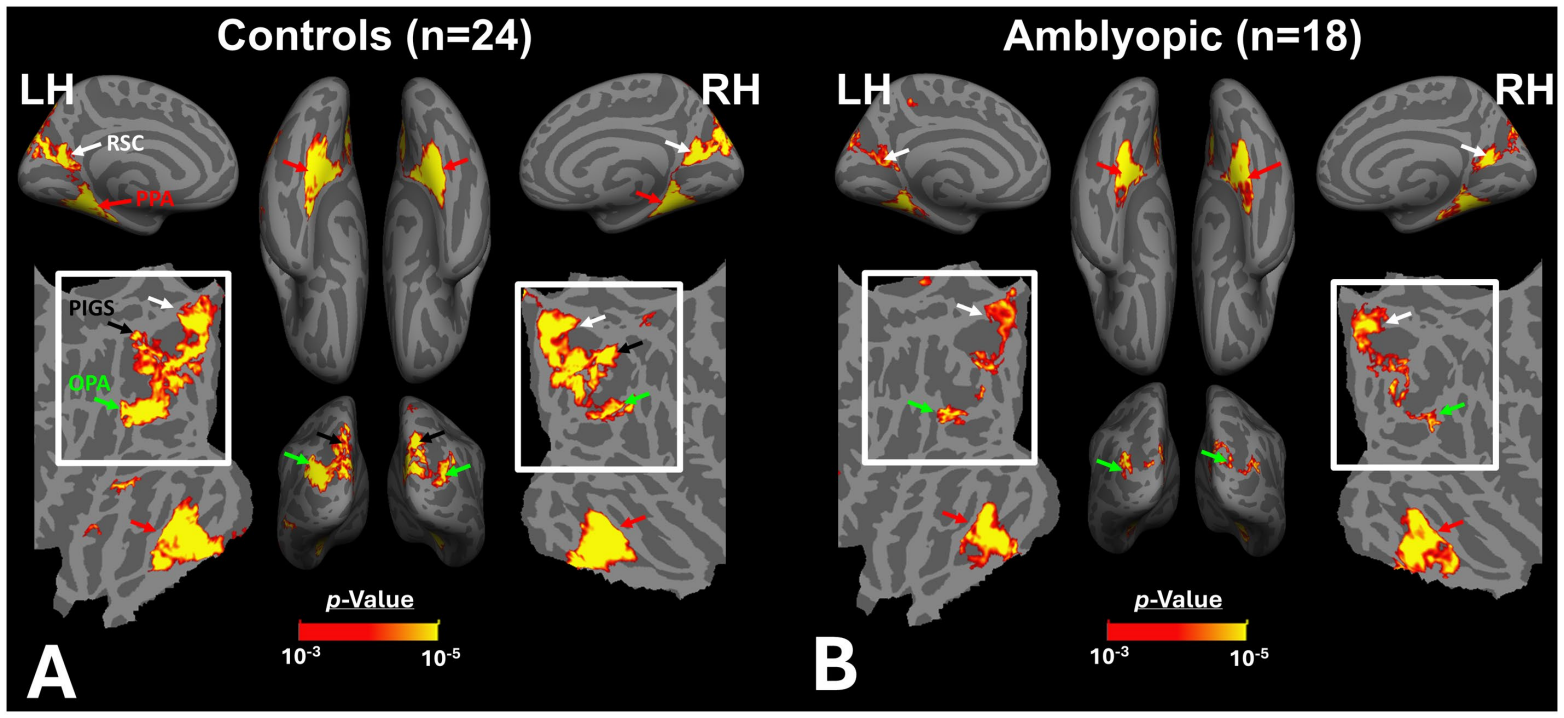
Group-averaged scene-selective activity in control **(A)** and amblyopic **(B)** participants. By measuring the response to “scene>face” contrast, we located areas PPA, RSC, OPA and PIGS (indicated by red, white, green and black arrowheads, respectively) for controls. In amblyopic individuals, we detected the same overall scene-selective activity pattern. However, in amblyopic compared to control participants, we found a weaker scene-selective activity within the posterior intraparietal gyrus (see also Figure 3). Both group-averaged activity maps were calculated based on random-effect analyses and were overlaid on a common brain template (fsaverage). The white inset indicates the occipito-parietal region in which PIGS, OPA and RSC are located. PPA: Parahippocampal Place Area; RSC: Retrosplenial Cortex; OPA: Occipital Place Area; PIGS: Posterior Intraparietal Gyrus Scene-Selective Area.

**Figure 3:**
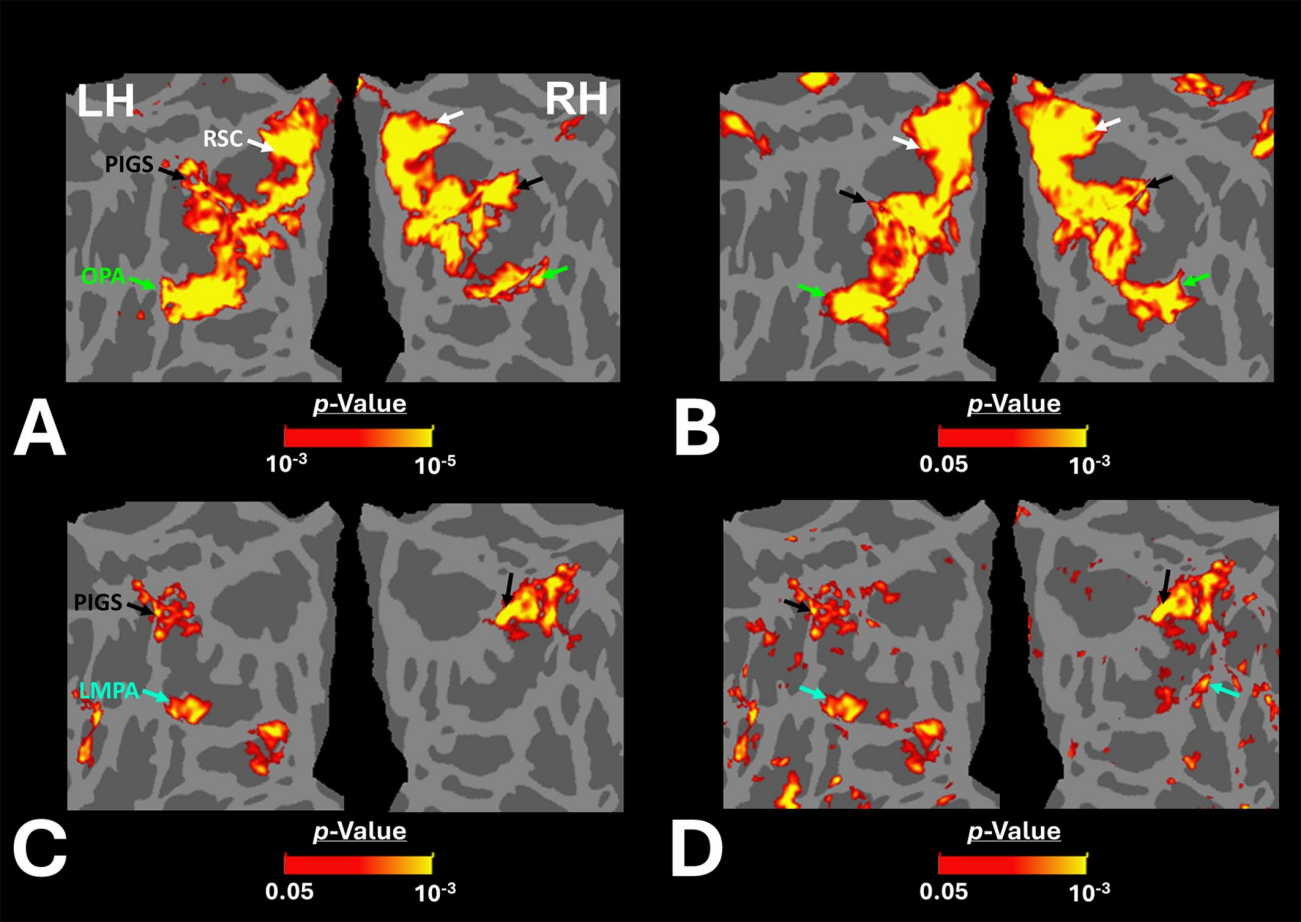
Group-averaged scene-selective activity in control and amblyopic participants across the occipito-parietal region. **(A,B)** show the activity maps in controls and amblyopic individuals, respectively. For amblyopic individuals, the activity map is generated based on lower threshold levels. Despite using those more liberal thresholds, the scene-selective activity within the posterior intraparietal gyrus appeared to be weaker in amblyopic compared to control participants. **(C,D)** show the between-group scene-selective activity differences, with and without correction for multiple comparisons, respectively. Consistently, we found bilateral scene-selective activity difference within the posterior intraparietal region. In Panel D, beyond the sensory scene-selective areas, we also noticed a bilateral activity difference within the LPMA region, as reported previously using conventional fMRI (Steel et al., 2021; Steel et al., 2023). LPMA: lateral place memory area. Other details are similar to those in Figure 2.

**Figure 4:**
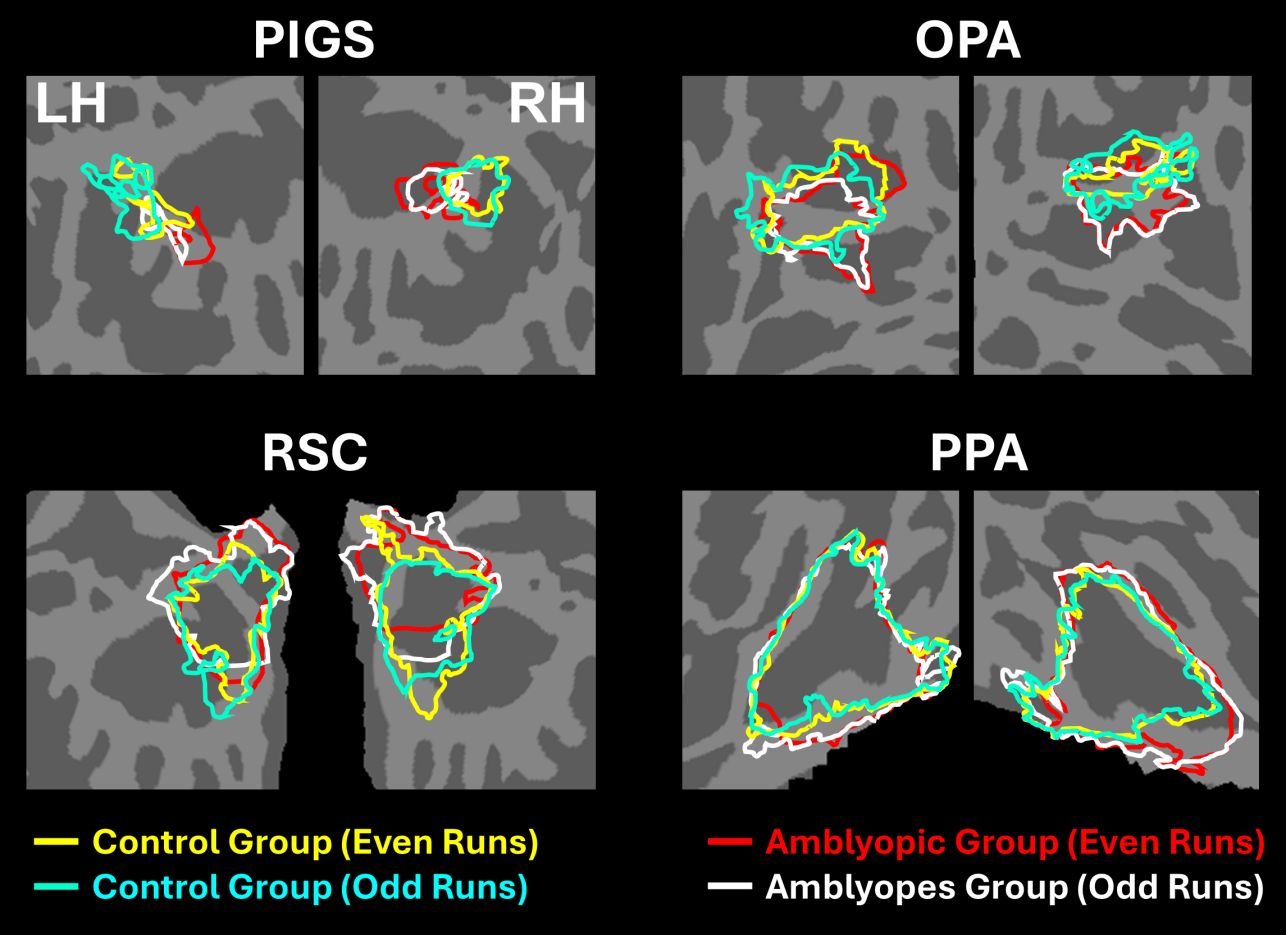
Consistency of localization in scene-selective areas across odd and even runs. In amblyopic and control groups, scene-selective areas were detected across odd and even runs. PIGS in amblyopic individuals is on average located more ventrally compared to controls, whereas RSC, OPA and PPA are located in a similar location between amblyopic and control participants. This difference in PIGS location was detected during odd and even runs, without any apparent differences.

Figures 2A,B shows the scene-selective activity maps generated by contrasting the evoked response to scene vs. face stimuli in control and amblyopic participants, respectively. In both groups, we were able to identify the PPA, RSC and OPA, without any apparent differences in the location of these areas between control and amblyopic groups. In controls, we were also able to detect area PIGS, close to the posterior border of the parieto-occipital sulcus within the intraparietal gyrus (Figures 2A, 3A) (Kennedy et al., 2024). In the group-averaged maps from the amblyopic participants, PIGS was detectable only when the threshold was lowered to *p* < 0.05 (Figure 3B). Even in these low-threshold maps, the center of PIGS appeared to be located more ventrally in amblyopic participants compared to the control group. This difference was also detectable when we generated the PIGS label based on the group-averaged activity maps evoked independently during odd and even runs (Figure 4).

Figure 3C shows the vertex-wise map of activity difference between controls and amblyopic individuals, after correction for multiple comparisons. In both hemispheres, we found stronger scene-selective activity within PIGS in the controls compared to amblyopic participants. Besides PIGS, we also found a significant between-groups activity difference in the anterior intraparietal gyrus, on the opposite side relative to PIGS, and posteriorly relative to the medial and superior temporal sulci. Named lateral place memory area (LPMA), this region is expected to be involved in place memory retrieval (Steel et al., 2021; Steel et al., 2023). This latter activity appeared to be stronger in the left compared to the right hemisphere. However, the same pattern of activity was also detectable in the right LPMA region, when analyzed without correction for multiple comparisons (Figure 3D).

**Figure 5:**
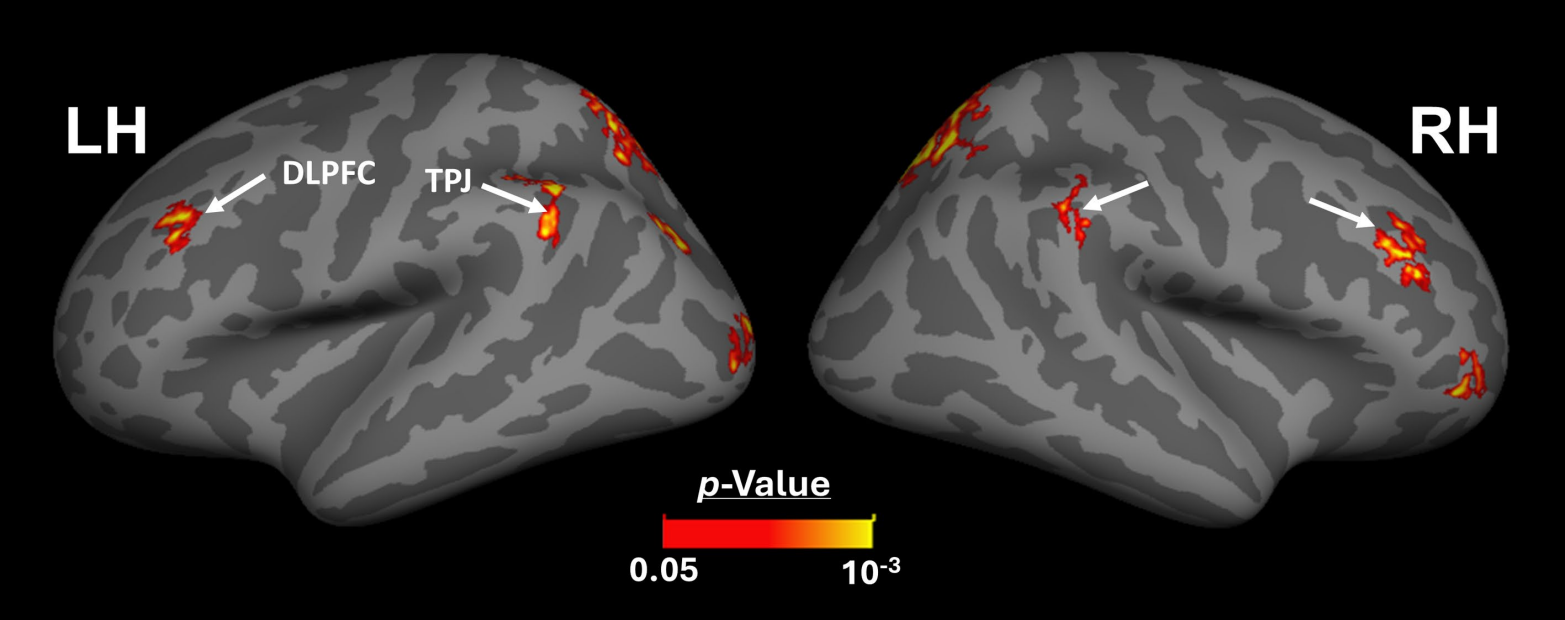
Between-group activity differences outside the visual areas. Beyond the visual areas, we found bilateral scene-selective activity differences between control and amblyopic individuals in areas TPJ and DLPFC. This result suggests that the impact of amblyopia on the response to the ‘scene>face’ contrast may extend to association brain areas. DLPFC: Dorsolateral Prefrontal Cortex; TPJ: Temporal Parietal Junction. The other details are similar to Figure 2.

Beyond the sensory areas, we also found bilateral activity differences within the temporal parietal junction (TPJ) and dorsolateral prefrontal cortex (DPFC), two regions that are expected to be involved in attention control and decision making (Figure 5). Importantly, these activity differences were detected even though participants were not instructed to do any scene-related tasks such as memory recall (Steel et al., 2021; Steel et al., 2023) or spatial comparison (Nasr and Tootell, 2012b; Nasr et al., 2013). Considering that the same areas (i.e., LPMA and TPJ) also did not appear within the scene-selectivity maps in either control or amblyopic groups (Figures 2, 3), the significant difference between the groups was most likely due to subthreshold responses within these regions. Due to such uncertainties, they were excluded from the rest of the data analysis (see Discussion).

#### 3.3.3 Localization of scene-selective areas in individual participants

**Figure 6:**
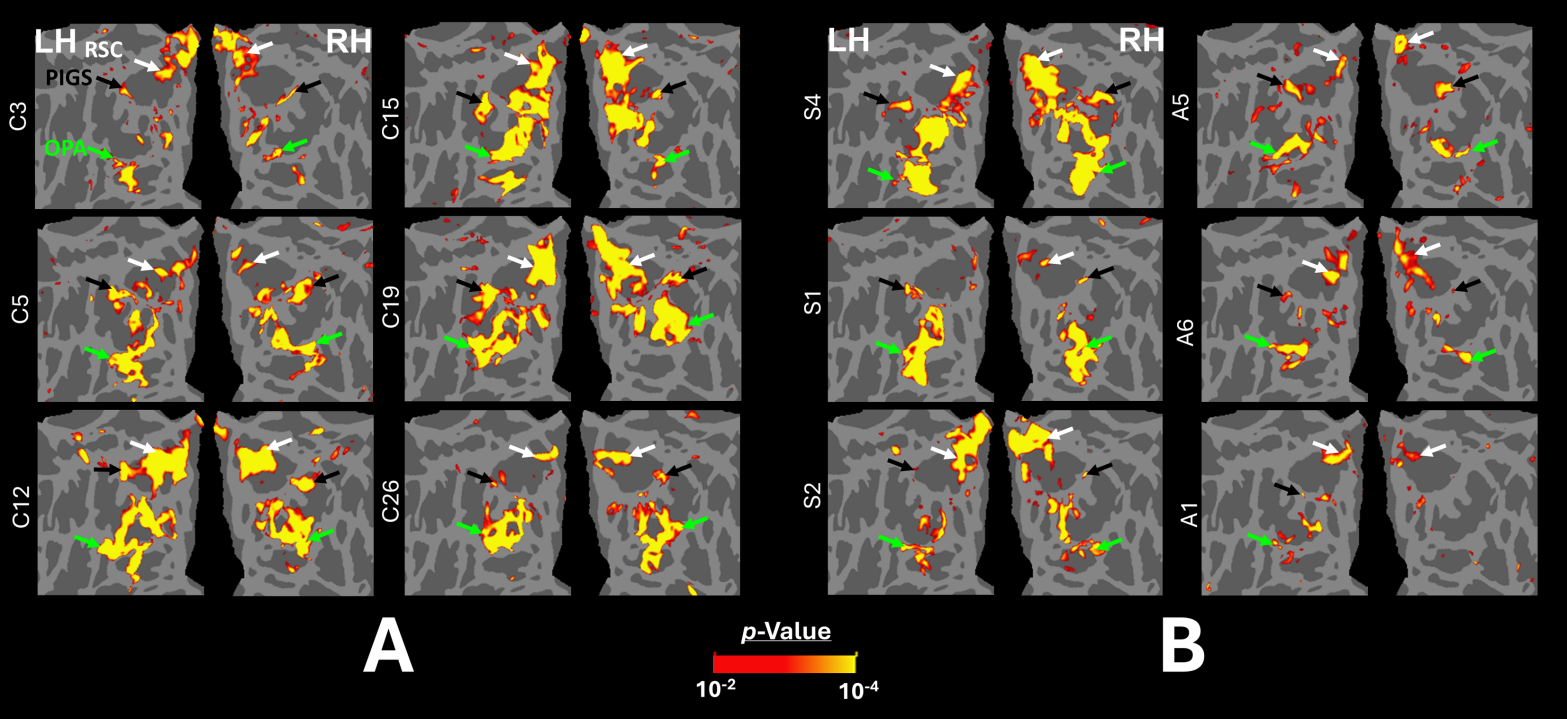
Localization of scene-selective areas within the occipito-parietal cortex across controls and amblyopic individuals. **(A)** shows the data from the left and right hemispheres of 6 control participants. **(B)** shows the data from 3 exemplar strabismic (left) and 3 exemplar anisometropic (right) individuals that show strong (top), medium (middle) and weak (bottom) scene-selective activity within the posterior intraparietal region (black arrow). For all participants, the activity maps were overlaid on a common brain template (fsaverage) to facilitate the comparison across individuals. PIGS, RSC, and OPA are indicated with black, white and green arrowheads.

In all participants, including the amblyopic individuals, we were able to localize PPA, RSC, and OPA bilaterally, at a threshold level of *p* < 0.01 (Figure 6). PIGS was also detected in all tested control participants, bilaterally. However, in 5 amblyopic participants, we could not detect PIGS either bilaterally (3 individuals) or unilaterally (2 individuals, contralateral relative to the amblyopic eye in both cases) at this threshold level. When normalized relative to the size of the whole cortex, the average size of PIGS (but not the other scene-selective areas) was significantly smaller in amblyopic individuals compared to controls (Table 4).

**Table 4:** Normalized^a^ size of scene-selective areas in amblyopic and control participants.

Area	Amblyopic LH	Amblyopic RH	Control LH	Control RH	*F*-value	*p*-value with correction
PIGS	0.14% ± 0.14%	0.14% ± 0.13%	0.28% ± 0.14%	0.23% ± 0.12%	10.12	0.01**
OPA	0.87% ± 0.51%	0.75% ± 0.48%	1.13% ± 0.48%	0.93% ± 0.51%	0.63	>0.99
RSC	0.35% ± 0.36%	0.40% ± 0.35%	0.44% ± 0.27%	0.45% ± 0.45%	2.34	0.52
PPA	1.33% ± 0.46%	1.17% ± 0.42%	1.41% ± 0.43%	1.30% ± 0.49%	0.65	>0.99

a All values are measured in percentage relative to the overall size of the cortex.

**: *p* ≤ 0.01.

#### 3.3.4 The amplitude of the scene-selective activity across amblyopic and control participants

**Figure 7:**
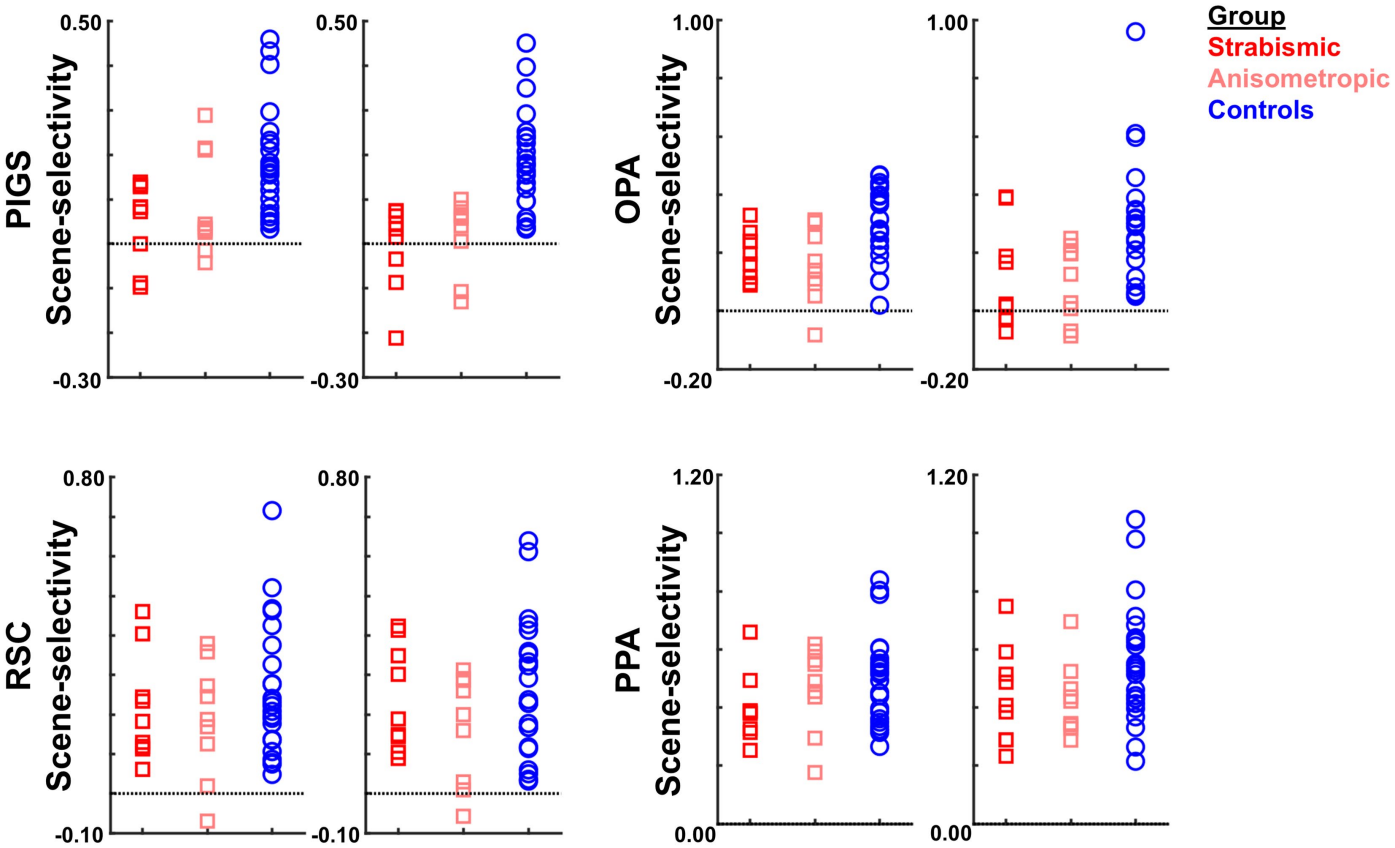
The level of scene-selective activity (scenes – faces) was measured across areas PIGS, OPA, RSC, and PPA, defined based on two separate previously published datasets (Nasr and Rosas, 2016; Steel et al., 2023). Across all ROIs, we found a significant difference only in the level of scene-selective activity between amblyopic individuals vs. controls in area PIGS. The difference between strabismic and anisometropic individuals remained non-significant across all tested ROIs. For each area, the left and right panels show the activity measured within the left and right hemispheres, respectively. In each panel, each point represents data from one individual participant.

Figure 7 shows the amplitude of the scene-selective (scene vs. face) response, evoked within PIGS, OPA, RSC, and PPA. The targeted ROIs were determined independently based on a different group of participants (see Methods). The results of ROI analysis, based on the ROIs defined independently from a different group of subjects, and application of one-way repeated measures ANOVAs showed a significantly weaker scene-selective activity in PIGS for amblyopic individuals (irrespective of amblyopia sub-type) compared to controls (F(40, 1) = 18.97, *p* < 10^−3^; corrected for multiple comparisons), without a significant group × hemisphere interaction (F(40, 1) = 5.25, *p* = 0.11). Application of the same test to the measured activity within areas OPA, PPA, and RSC did not yield any significant effect of group and/or group × hemisphere interaction (Table 5). A separate ANOVA did not show any significant differences between the scene-selective activity measured in the strabismic vs. anisometropic individuals (*p >* 0.16).

**Table 5:** Between-groups (amblyopic vs. control individuals) differences in the level of scene-selective activity based on the ROIs from independent datasets.

Area	Group^a^		group × hemisphere	
	F	*p*	F	*p*
PIGS	18.97	10^−3^ **	5.25	0.11
OPA	5.94	0.08	1.20	>0.99
RSC	2.09	0.64	<10^−3^	>0.99
PPA	1.66	0.81	1.04	>0.99
V6	2.38	>0.99	0.26	>0.99

a The effect of group was tested between controls vs. amblyopic individuals, irrespective of their amblyopia sub-type.

**: *p* ≤ 0.01.

**Figure 8:**
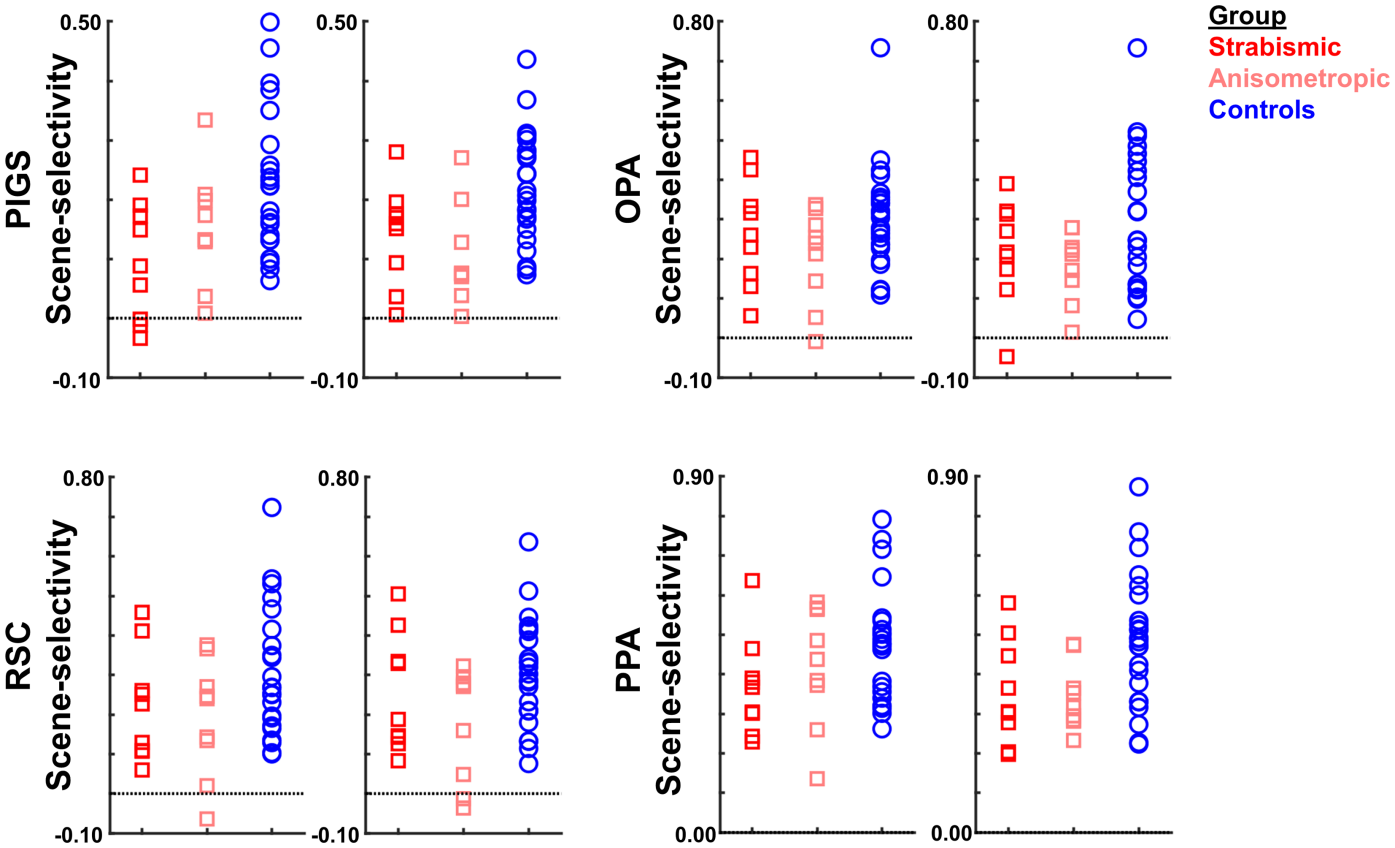
The level of scene-selective activity (scenes – faces) was measured across areas PIGS, OPA, RSC, and PPA. Across all ROIs, we found a significant difference only in the level of scene-selective activity between amblyopic individuals vs. controls in area PIGS. The difference between strabismic and anisometropic individuals remained non-significant across all tested ROIs. All details are similar to Figure 7.

It could be argued that the differential localization of the scene-selective activity within the posterior intraparietal gyrus may contribute to the detected between-groups difference. To test this possibility, we repeated our tests using a separate set of labels, generated for each group based on their own group-averaged activity map (see Methods). In this approach, to avoid logical circularity, the group-averaged activity maps based on the “odd” runs were used to localize the ROIs that were used to measure the response during “even” runs, and vice versa. As shown in Figure 8, the result of this analysis still showed a significantly weaker scene-selective activity in PIGS for amblyopic individuals compared to controls (F(40, 1) = 12.38, *p* = 0.01), without a significant group × hemisphere interaction (F(40, 1) < 0.01, *p* = 0.98). Here again, application of the same test to the measured activity within areas OPA, PPA, and RSC did not yield any significant effect of group and/or group × hemisphere interaction (Table 6). Moreover, a separate ANOVA did not show any significant differences between the scene-selective activity measured in the strabismic vs. anisometropic individuals (*p >* 0.45).

**Table 6:** Between-groups (amblyopic vs. control individuals) differences in the level of scene-selective activity based on the ROIs generated for the amblyopic and control groups based on their own fMRI activity.

Area	Group^a^		group × hemisphere	
	F	*p*	F	*p*
PIGS	12.38	0.01**	<0.01	>0.99
OPA	3.93	0.25	0.40	>0.99
RSC	3.78	0.24	0.05	>0.99
PPA	6.81	0.06	3.13	0.32

a The effect of group was tested between controls vs. amblyopic individuals, irrespective of their amblyopia sub-type.

**: *p* ≤ 0.01.

As a control analysis, we also measured the activity evoked within area V6 (see Methods). V6 was selected based on their proximity to area PIGS and its stronger response to scenes compared to objects (Sulpizio et al., 2020; Kennedy et al., 2024). Tests in V6 showed no significant scene-selective activity difference between amblyopic and control individuals (Table 5). Thus, among those regions that showed scene-selective activity, reduced activity in amblyopia was mainly limited to area PIGS. Notably, the intact activity within PPA, OPA, RSC and V6, rules out the possibility that this result is due to the impact of amblyopia on attention control and/or fixation steadiness (see Discussion).

#### 3.3.5 Predictability of VFQ-39 scores based on the scene-selective responses

**Figure 9:**
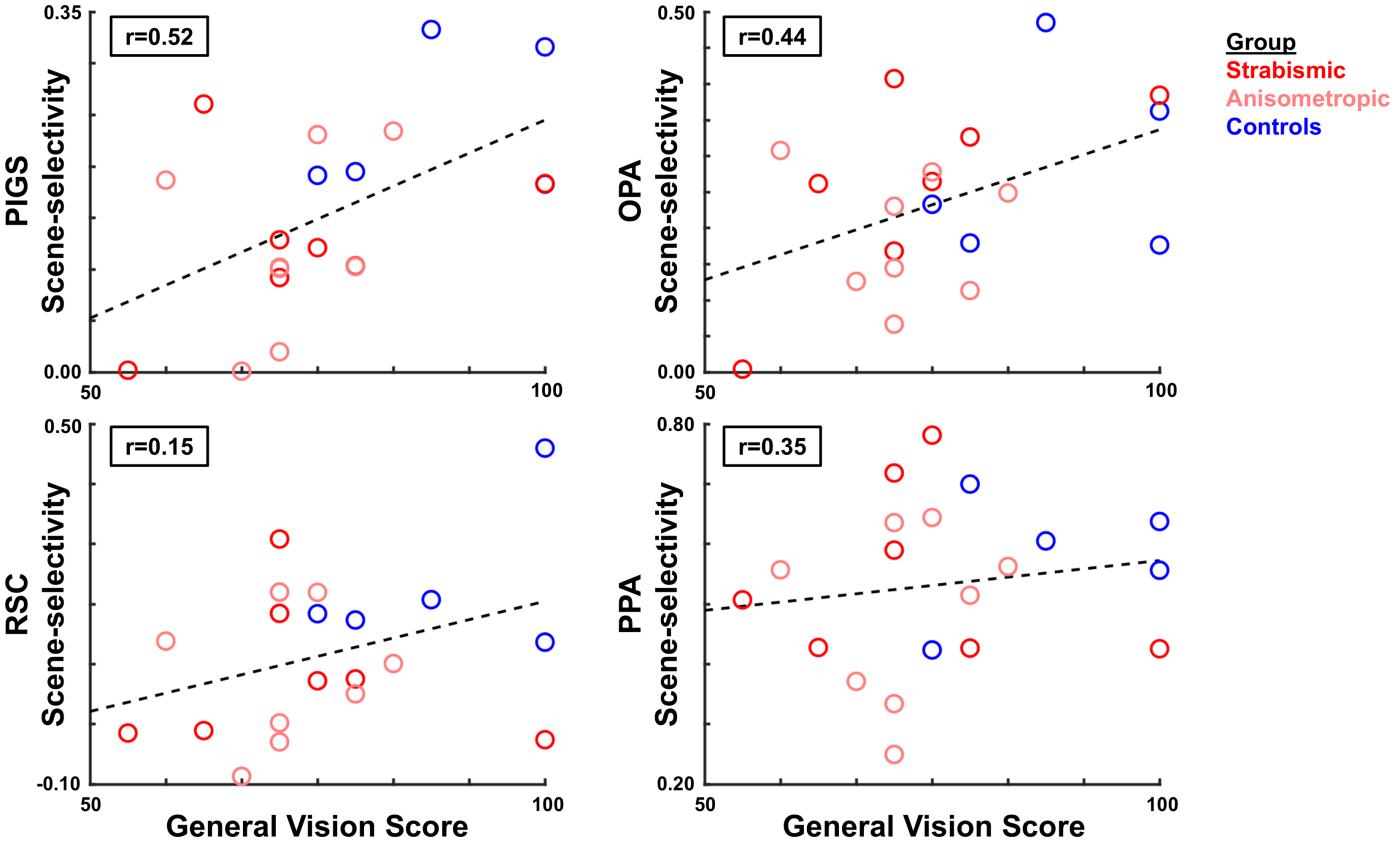
Correlation between the level of scene-selective activity and the reported score for general vision (VFQ-39 subscale). Across the scene-selective areas, we found a significant correlation only within areas PIGS and OPA. In each panel, each circle represents data from one individual participant, averaged over the two hemispheres. Groups are indicated by color.

For the twenty individuals who participated in Experiments 1 and 2 (Table 1), we checked whether the reported scores for general vision, distance activities, and peripheral vision (based on VFQ-39) correlated with the level of scene-selective area activities based on the fMRI results. Considering the consistency of the results between the two sets of the ROI labels for the scene-selective areas (see above), we limited our analysis to activity measured in those ROIs that were defined for each group based on their own (scene>face) activity response. Independent Pearson correlation tests showed a significant linear relationship between the reported score for general vision and the level of scene-selective activity within PIGS (df = 18, R^2^ = 0.28, *p* = 0.02), and OPA (df = 18, R^2^ = 0.20, *p* = 0.05) (Figure 9). General vision was not significantly correlated with activity in RSC or PPA (df = 18, R^2^ < 0.15*; p* > 0.08). Importantly, we did not find any significant correlations between measures of interocular visual acuity difference, binocular visual acuity, and stereoacuity with scene-selective activity across different ROIs (df = 18, R^2^ < 0.10*; p* > 0.20). These results suggest that the correlation between the scene-selective response and self-reported visual function reflects the higher-order visual functional deficits of amblyopia rather than those of discrete spatial or interocular disparity resolution. Here again, application of the same test just to the results from the amblyopic individuals did not yield any significant correlation between the VFQ scores and fMRI measurements (*p* > 0.10). More amblyopic individuals with a wide range of visual acuities are required to test this hypothesis more thoroughly in the future.

### 3.4 Experiment 3 – object-selective cortical responses

Experiment 3 tested whether the amblyopic participants also show a decreased object-selective activity, and if so, whether this reduction contributed to the observed decrease in the level of scene-selective activity in area PIGS. Accordingly, eighteen amblyopic individuals plus seventeen controls (selected from those who participated in Experiment 2 based on their willingness to continue the scans) were scanned to measure their brain activity in response to the binocular presentation of intact vs. scrambled objects (see Methods and Table 1).

#### 3.4.1 Head position stability

As in Experiment 2, we compared the level of head motion during scanning, between control vs. amblyopic participants. A t-test applied to the measured level of head motion did not yield a significant difference between the two groups (t(33) = 0.27, *p* = 0.80), suggesting that head position was comparable between the two groups. Nevertheless, as in Experiment 2, we included this nuisance covariate in all analyses, to eliminate residual effects of head motion.

#### 3.4.2 Group-averaged object-selective response

**Figure 10:**
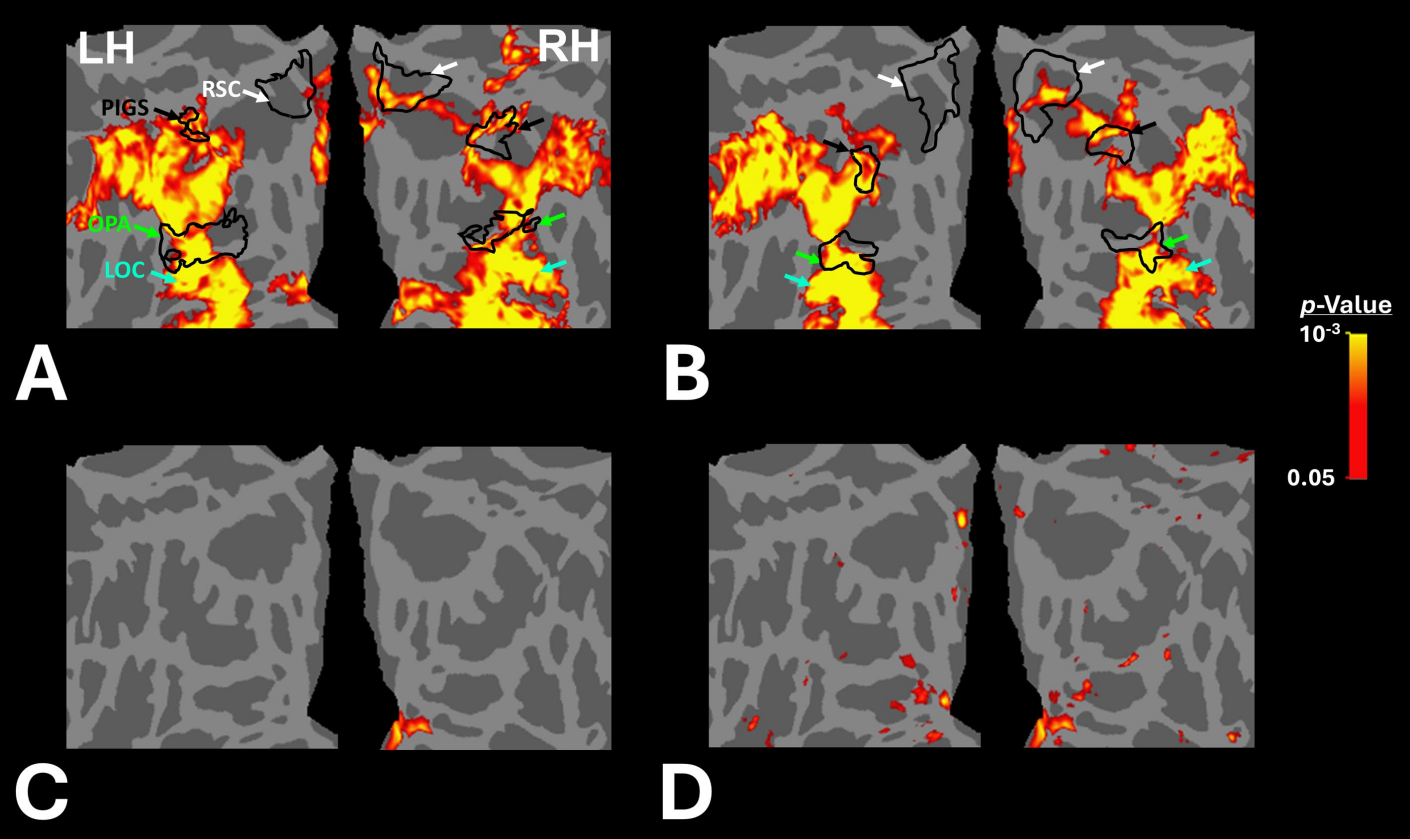
The group-averaged object-selective activity (intact object>scrambled object) in control and amblyopic participants across the occipito-parietal region. **(A,B)** show the activity maps in controls and amblyopic individuals, respectively. In contrast to the scene-selective activity map within the same region the overall pattern of object-selective activity appears to be comparable between the two groups, even within the posterior intraparietal region. In these panels, the location of areas LOC, OPA, PIGS and RSC are indicated by arrowheads. The borders of scene-selective areas are indicated by black lines (see also Figure 3). **(C,D)** show the between-groups object-selective activity differences, with and without correction for multiple comparisons, respectively. Here again, we did not find any significant difference between the two groups. LOC: Lateral Object-Selective Complex. The other details are similar to Figure 2.

In both groups, object-selective activity was detected within a large portion of the extra-striate visual cortex, including the scene-selective areas PIGS, OPA, RSC, and PPA, plus object-selective area LOC (Figures 10A,B). In contrast to scene-selective activity maps, here, the vertex-wise between-group comparison did not show any significant difference between amblyopic and control participants across the scene-selective areas (Figures 10C,D) and also within area LOC. Thus, object-selective responses were comparable between the two groups.

#### 3.4.3 The amplitude of the object-selective activity across the amblyopic and control participants

**Figure 11:**
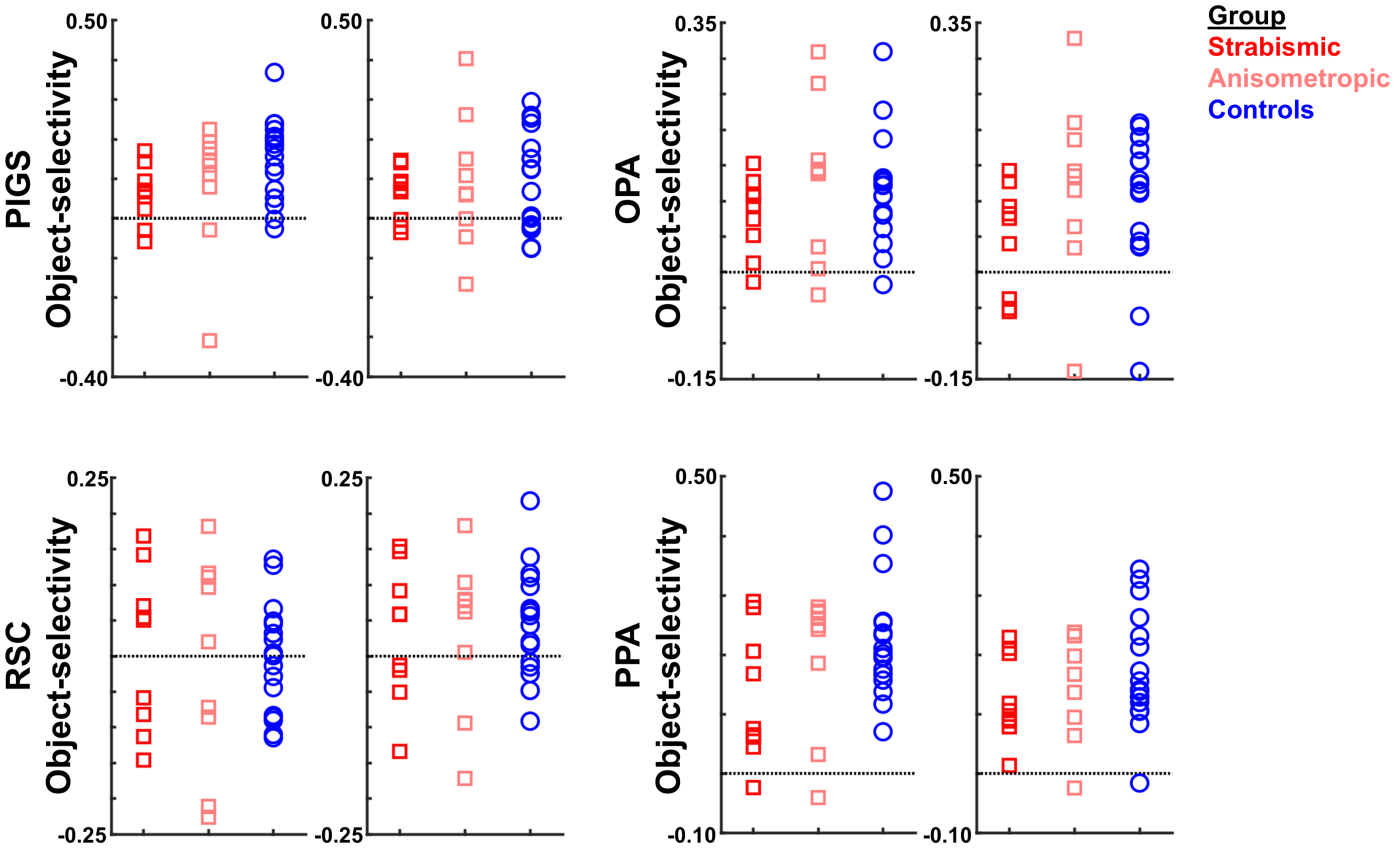
The level of object-selective activity (intact objects – scrambled objects) was measured across areas scene-selective areas PIGS, OPA, RSC, and PPA. Consistent with the activity maps, none of these areas showed any significant object-selective activity difference between amblyopic individuals and controls. All details are similar to Figure 7.

Within the scene selective areas, we further compared the object-selective response between amblyopic and control participants, using a more sensitive ROI-based analysis (Figure 11). Here again, we limited our ROIs to those that were defined for each group based on their own (scene>face) activity response. Consistent with the group-averaged activity maps, results of this test did not yield any significant effect of group (*p* > 0.72; corrected for multiple comparisons) and/or group × hemisphere interaction (*p* > 0.48). These results suggest that the impact of amblyopia on the activity within the scene-selective areas was limited to the evoked response to scenes, and this effect was not attributable to altered object processing. Here again, comparable object-selective activity between amblyopic and control groups suggests that, in the current stimulus presentation paradigm, the potential impact of amblyopia on fixation steadiness is unlikely to cause a decrease in the level of selective responses (see Discussion).

## 4 Discussion

The results of this study directly suggest that amblyopia impacts scene processing within PIGS, a scene-selective area located within the posterior intraparietal gyrus (Kennedy et al., 2024). We found that scenes (but not single objects) evoked weaker selective activity in PIGS (but not OPA, RSC and PPA) in amblyopic participants compared to controls. The correlation between scene-selective activity in this area and the self-reported general vision score – a subscale of the VFQ-39 quality-of-life questionnaire – underscores the critical role of higher-level visual areas in shaping how individuals with amblyopia perceive their overall visual world. However, the direct link between these two measurements (i.e., general health (behavior) vs. brain activity [fMRI measurements)] needs to be assessed carefully in the future.

### 4.1 Amblyopia impacts on higher-order visual processing

Originally, amblyopia studies were mainly focused on area V1 because neurons within this cortical area show strong ocular preference (Hubel and Wiesel, 1962). According to early studies, amblyopia (especially in more severe forms) was associated with a decrease in the number of neurons that respond preferentially to the amblyopic eye (Crawford and Von Noorden, 1979; Crawford et al., 1996; Smith et al., 1997b; Kiorpes et al., 1998). Later studies showed that amblyopia is also associated with a decrease in the number of disparity-selective neurons that are distributed over the extrastriate visual areas such as V2 (Nakatsuka et al., 2007; Bi et al., 2011).

By comparing the response to stimulation of the fellow and amblyopic eye, multiple neuroimaging studies have shown strong response to the fellow eye stimulation in multiple visual area including V4, V8 and LOC (Muckli et al., 2006; Nasr et al., 2025). Moreover, consistent with our findings, Lerner et al., have also shown evidence for reduced face-selective (but not scene-selective) activity within the fusiform gyrus when face and scene stimuli were presented to the amblyopic rather than the fellow eye (Lerner et al., 2003). But they did not extend their tests to the intraparietal area in which PIGS is located, and they did not compare the evoked responses between individuals with normal and amblyopic vision when the stimuli are presented binocularly.

Our current results extend these findings by suggesting that the impact of amblyopia on the development of the visual cortex extends well into the higher-level visual areas that respond selectively to scenes. For one thing, stimuli were presented binocularly rather than monocularly to minimize the potential impacts of visual acuity difference between amblyopic individuals and controls. Moreover, the stimuli did not contain any stereo cues and did not induce any coherent motion – two visual features whose encoding is impaired in amblyopic participants (McKee et al., 2003; Aaen-Stockdale et al., 2007; Aaen-Stockdale and Hess, 2008; Levi et al., 2015). Nevertheless, deficient PIGS activity was evoked by scenes, but not non-scene objects. Together, our findings indicate that amblyopia selectively impacts scene processing within this region.

### 4.2 Why PIGS but not the other scene-selective areas?

PIGS is located within the posterior intraparietal gyrus, an area populated with motion- and stereo-selective sites (Tootell et al., 2022; Kennedy et al., 2023). It also contributes to ego-motion encoding within naturalistic environments, a perceptual process that relies heavily on depth, motion coherence (optic flow), and ego distance estimation. Previous psychophysical studies have shown that all these visual functions (i.e., depth, motion coherency and ego distance perception) are, at least to some extent, impaired in amblyopic individuals (McKee et al., 2003; Melmoth and Grant, 2006; Aaen-Stockdale et al., 2007; Aaen-Stockdale and Hess, 2008; Carlton and Kaltenthaler, 2011; Grant and Moseley, 2011; Levi et al., 2015; Ooi and He, 2015). Thus, in the absence of normal visual input, experience-dependent development of PIGS could be disrupted in amblyopia. Our findings of decreased scene-selective activity within PIGS in amblyopic individuals support a neurodevelopmental component to abnormal amblyopic scene processing. Such a developmental disorder may even worsen over time in the absence of engagement in visually guided tasks that rely on ego-motion (Sá et al., 2021; Harrington et al., 2023), which also reduces the level of feedback to these regions.

In contrast to PIGS, PPA and RSC do not respond to ego-motion (Hacialihafiz and Bartels, 2015; Kamps et al., 2016; Jones et al., 2023; Kennedy et al., 2024). Rather, PPA and RSC are involved in scene recognition (Epstein et al., 2007; Park and Chun, 2009) and layout representation (Wolbers et al., 2011), respectively. Area OPA also appears to respond to simpler forms of motion, whose encoding is less affected by amblyopia. However, considering the functional connection between scene-selective areas (Baldassano et al., 2013; Nasr et al., 2013; Baldassano et al., 2016), it may be possible to see more extended between-groups differences during complex, scene-related tasks that rely simultaneously on multiple visual cues.

### 4.3 Weaker input from the amblyopic eye is not the sole cause of scene-selective activity decrease in PIGS

By relying on their fellow eye, amblyopic participants usually show comparable binocular visual acuity relative to normally sighted individuals. However, it can still be argued that stimulation of the amblyopic eye likely evokes a weaker visual response, compared to either their fellow eye or the non-dominant eye in controls (Conner et al., 2007; Dorr et al., 2019; Nasr et al., 2025). Considering this, one may suggest that the activity decrease in PIGS is due to a weaker bottom-up input from the earlier visual areas to PIGS.

Two key results from our study argue against this explanation. First, intact scene-selective activity in other areas, including OPA, PPA, RSC, and V6, suggests that decreased visual input may not be the sole reason for this phenomenon. Notably, the impact of decreased visual input from the amblyopic eye is expected to be stronger on OPA that contributes to earlier stages of scene processing compared to PIGS. Specifically, OPA overlaps with visual areas V3A/B and V7 (IPS0) and responds to simple visual cues such as translational motion (Nasr et al., 2011; Silson et al., 2016), whereas PIGS is located adjacent to areas IPS2-4, and only responds to more complex visual cues such as optic flow caused by ego-motion in naturalistic scenes but not random dots (Kennedy et al., 2024). However, contrary to this expectation, our results showed that the between-groups scene-selective activity difference was mostly limited to PIGS.

Second, we found that, in all examined regions (including PIGS), the activity evoked by non-scene objects was not impacted by amblyopia, as would be expected if reduced visual gain drove differences in these higher-order regions. Thus, decreased visual input from lower-level visual areas cannot explain the weaker scene-selective response in PIGS.

### 4.4 The potential contribution of lower-level features

Previous studies have shown that scene-selective areas respond selectively to various lower-level features, including high spatial frequency features (Rajimehr et al., 2011; Zeidman et al., 2012; Kauffmann et al., 2015), cardinal (i.e., horizontal and vertical) orientations (Nasr and Tootell, 2012a), and rectilinearity (Nasr et al., 2014). Our face and scene stimuli were designed to rule out the potential impact of higher spatial frequency features in the evoked scene-selective response (see Methods). Thus, the known impact of amblyopia on the encoding of higher spatial frequencies (Hess et al., 2009; Farivar et al., 2011) could not be responsible for the between-groups difference in the level of PIGS response. However, we cannot rule out the possibility that PIGS activity is selectively driven by other lower-level visual features that are impacted by amblyopia. Notably, our understanding of PIGS function and stimulus selectivity remains very limited. Further studies are needed to shed light on the neuronal mechanisms of visual processing in this region.

### 4.5 Potential contribution of attentional deficits and fixation instability in amblyopia

Degraded visual attention in amblyopia has been previously reported by others (Ho et al., 2006; Hou et al., 2016; Verghese et al., 2019). In this study, we reduced the influence of attention on the level of scene-selective responses by instructing the participants to perform an orthogonal task (i.e., detection of color changes in the fixation spot). Nevertheless, it might be argued that uncontrolled attentional demand contributed to the between-groups activity difference that we found in PIGS. However, if this was the case, we would have expected to see the same effect in the other scene selective areas, such as PPA—in which attention to scenes increases the level of scene-selective activity (O'craven et al., 1999; Nasr and Tootell, 2012b; Baldauf and Desimone, 2014).

The same is also true for the potential impacts of fixation instability in amblyopia. Specifically, in amblyopic individuals, the amblyopic eye fixation stability is poorer compared to the fellow eye (Schor and Hallmark, 1978; Subramanian et al., 2013; Chung et al., 2015). This phenomenon is specifically more apparent in strabismic individuals contributing to a decrease in the visual acuity of the amblyopic eye (Chung et al., 2015). This phenomenon is expected to cause a non-selective decrease in the level of visually evoked activity in response to high spatial frequency stimuli when presented to the amblyopic rather than the fellow eye. However here, we only found a selective activity decrease in PIGS but not the other scene-selective areas. Moreover, the level of object-selective activity in PIGS remained equivalent between amblyopic and control groups, suggesting that the impact of amblyopia on PIGS function is mostly limited to the scene processing within this region.

Thus, in the absence of any significant activity decrease in the other scene-selective areas, and with comparable object-selective activity between the two groups, the potential impacts of amblyopia on attention and fixation stability are unlikely to be the cause of the decreased scene-selective activity in PIGS.

### 4.6 Monocular vs. binocular visual stimulation

To detect the impact of amblyopia, neuroimaging and electrophysiological studies typically (though not always) rely on comparing the activity evoked by stimulation of the amblyopic eye vs. the fellow eye (e.g., see Lerner et al., 2003). We avoided this approach primarily because scene perception is considered a binocular task for individuals with normal vision. Although it has been shown that scene perception impairments in amblyopic individuals are equally detectable under both monocular and binocular conditions (Mirabella et al., 2011), comparing the activity evoked by monocular visual stimulation between individuals with normal and amblyopic vision may undermine the between-group differences. However, this approach may still help clarify whether the impact of amblyopia on scene-selective activity is restricted to PIGS or also involves other brain regions.

### 4.7 Amblyopia impacts outside the occipito-parietal cortex

Our experimental design did not instruct the participants to explicitly categorize the stimuli into “scenes vs. non-scenes,” or to discriminate differing scene stimuli. In the absence of such tasks, we limited our analysis to activity evoked within the sensory visual areas, even though we reported multiple cortical sites in association brain area, including TPJ and DLPFC, in which we found between-group differences in the level of scene-selective response. The existence of these more anterior cortical sites suggests that amblyopia impacts may extend well beyond the sensory regions into areas of the association cortex that control different aspects of the human behavior, from attention control (Corbetta et al., 2000; Shulman et al., 2007) to perceptual decision-making (Heekeren et al., 2004; Philiastides et al., 2011). Such an extension may become even more apparent when participants are involved in an active scene-related task (e.g., navigation).

### 4.8 Self-reported measures vs. psychophysical tests

We administered the VFQ-39 questionnaire to determine whether our amblyopic participants also reported difficulties with near and distance vision, as suggested by previous QoL studies (Kumaran et al., 2019; Randhawa et al., 2023). While VFQ-39 assesses a broad range of daily activities, it does not identify the underlying perceptual impairments that contribute to the lower scores observed in amblyopic individuals compared to controls. To address these gaps, more direct, parametrized psychophysical tests are needed to uncover the specific impairments responsible for these difficulties.

That said, it is often challenging to link a single psychophysical measure (e.g., visual acuity) to an individual’s performance in complex tasks, such as navigation. Instead, a comprehensive battery of behavioral tasks is required to evaluate all aspects of a single (but multi-dimensional) cognitive task. Even in such cases, it remains unclear to what extent a specific task contributes to overall QoL. Therefore, psychophysical tests and QoL assessments appear to be complementary, each offering unique insights into the impact of amblyopia.

### 4.9 Limitations

Amblyopia influences many aspects of visual perception, from stereopsis to motion coherency. To distinguish the impact of amblyopia on scene perception and the underlying neuronal processing from its impact on depth and motion coherency encoding, we designed a paradigm based on using 2D stimuli that did not induce any coherent motion. While this paradigm serves to isolate the impact of amblyopia on scene-selective processing, our approach may underestimate the impact of amblyopia on scene perception as experienced by amblyopic individuals in their daily lives and weakens the correlation between the level of evoked brain activity and self-reported visual functional scores. However, the correlation between the scene-selective activity and general vision score relationships in PIGS and OPA (Figure 9) argues that the relationship is maintained across the functional spectrum in our sample.

Moreover, natural scene perception relies on input from the peripheral visual field (Levy et al., 2001; Hasson et al., 2002; Levy et al., 2004; Larson and Loschky, 2009). Thus, our restriction of visual stimuli to the central 20 degrees of the visual field may miss important contributions of more peripheral scene cues in a more immersive environment. However, central stimulation mitigates potential confounding by differences in binocular visual field sensitivity (perhaps more often seen in strabismus) and potential impairments in distributing spatial attention over a larger visual field.

Lastly, we used a limited number of scene images in our tests mainly because testing larger stimulus sets required a much larger data acquisition time. However, in a separate study, we have already shown that the selectivity of PIGS response is detectable based on a wide range of scenes (including indoor and outdoor scenes) without any apparent dependency between the location of center of PIGS activity and the type of scene stimuli (Kennedy et al., 2024). Moreover, the PIGS is detectable based on either ‘scene vs. face’ and ‘scene vs. object/face’ contrasts. Considering these effects, we expect the amblyopia impact on PIGS activity to be also detectable independent from the type of presented scenes and/or the stimulus contrast.

## 5 Conclusion

Our results show that the impact of amblyopia extends beyond the early retinotopic visual areas into cortical regions involved in scene processing. The results also highlight the likelihood that amblyopia affects the function of association brain regions such as LPMA, TPJ and DLPFC. Future studies employing more realistic, immersive stimuli (and tasks) that better resemble daily visual experiences could more comprehensively highlight the functionally relevant neural consequences of amblyopia in higher order visual areas.

## Data Availability

The raw data supporting the conclusions of this article will be made available by the authors, without undue reservation.
